# Atrial Esophageal Fistulas: A Case Series Demonstrating Three Distinct Operative Approaches with Favorable Outcomes

**DOI:** 10.1016/j.atssr.2025.03.017

**Published:** 2025-04-08

**Authors:** Eric Klipsch, Maxwell F. Kilcoyne, Gwyneth Bradley, Lucas Witer, Ian C. Bostock, Barry C. Gibney, Kathryn E. Engelhardt

**Affiliations:** 1 Division of Cardiothoracic Surgery, Medical University of South Carolina, Charleston, South Carolina; 2 Division of Thoracic Surgery, Miami Cancer Institute, Baptist Health South Florida, Miami, Florida

## Abstract

**Background:**

Atrial esophageal fistula (AEF) is a rare complication of atrial ablation procedures. Because of the low incidence and associated morbidity and mortality, there is no consensus on optimal treatment. We present our experience with 4 patients, each with a different management strategy.

**Methods:**

Retrospective chart review of 4 patients treated for AEF at a single tertiary care center was performed. Patients were treated between March 2020 and April 2024.

**Results:**

Three patients underwent surgical repair of the AEF and 1 underwent a combination of endoscopic and percutaneous approaches. Two of the surgical patients underwent right thoracotomy, while the third was approached via median sternotomy on cardiopulmonary bypass followed by a right thoracotomy. The 3 patients who underwent surgical repair are alive and tolerating oral intake. The patient who underwent endoscopic/percutaneous AEF repair had a considerably higher surgical risk at baseline. The patient recovered from the initial interventions and was discharged home but represented with AEF progression and subsequently expired secondary to neurologic sequalae.

**Conclusions:**

For AEF, open surgical management with or without the use of cardiopulmonary bypass has historically been the first-line treatment. Although successful management of AEF with endoscopic treatment has been documented in the literature, only surgical repair was successful in this case series. This series suggests that surgical management should be pursued for all patients except those with highly prohibitive surgical risk.

**In Short**

▪ Physicians should have a high clinical suspicion for atrial esophageal fistula formation in patients with recent atrial ablation who present with neurologic changes or bacteremia.
▪ Prompt surgical intervention should be the first-line treatment for those who are fit to undergo an operation with or without the use of cardiopulmonary bypass.
▪ Esophageal stenting should be reserved for those patients with prohibitive operative risk.

An atrial esophageal fistula (AEF) is a rare complication following left atrial ablation, occurring in <0.1% to 0.25% of cases, and, if left untreated, it can lead to significant morbidity and mortality.^1^^,^^2^ AEF development can rapidly progress to devastating neurologic and hemodynamic consequences.^3^ Open surgical repair with or without the use of cardiopulmonary bypass (CPB) has historically been the first-line treatment while endoscopic techniques have become an emerging modality.^3^^,^^4^ We present 4 patients treated for an AEF at our institution with different management strategies.

## Patients and Methods

This study was approved by the institutional review board at the Medical University of South Carolina, with a waiver of informed consent (Pro000135899, approved on April 2, 2024). However, given the nature of this report, individual informed patient consent was obtained. Patients with the diagnosis of an AEF at our institution were retrospectively reviewed. An overview of the 4 patients’ clinical characteristics and outcomes are displayed in Supplemental Table 1.

## Results

### Patient 1

**Figure 1:**
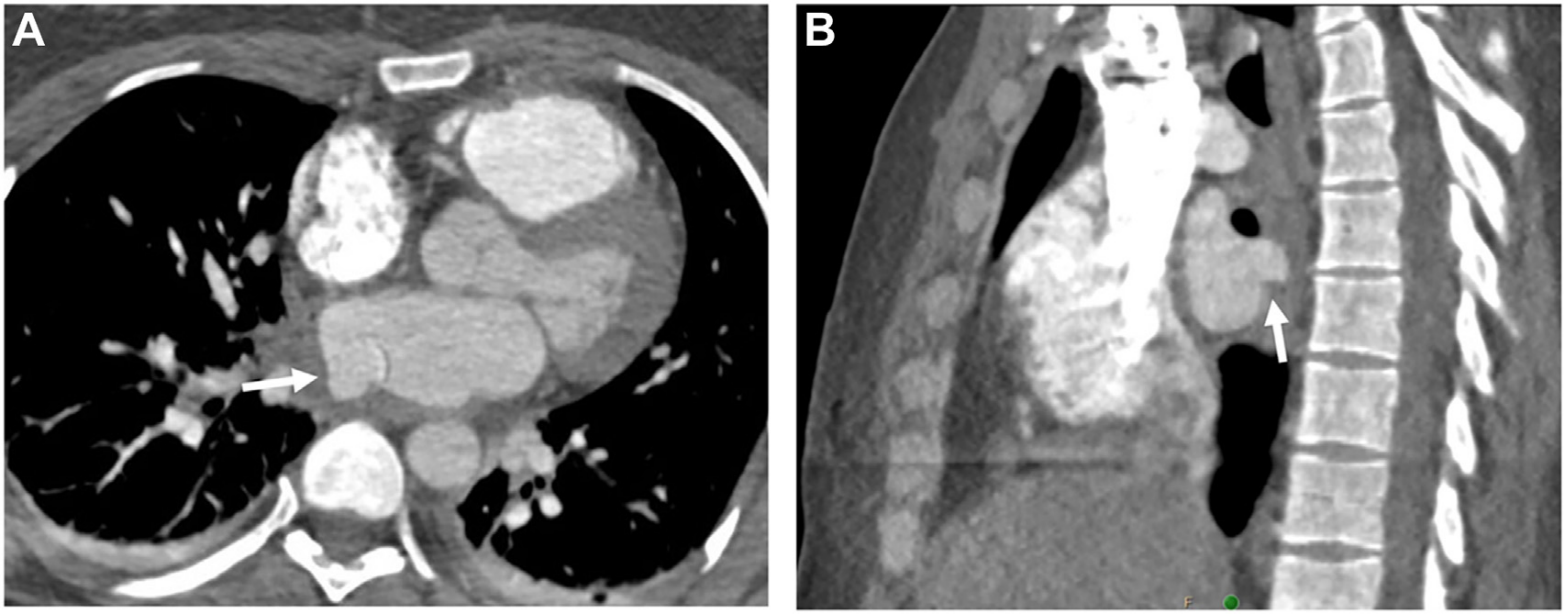
Axial (A) and sagittal (B) views of cardiac computed tomography angiograph demonstrating the left atrial pseudoaneurysm (arrow) and adjacent intrapericardial air.

A 47-year female patient with history of gastroesophageal reflux disease was admitted 2 weeks after atrial cryoablation and radiofrequency ablation with neurologic changes and imaging demonstrating multifocal septic emboli. Her ablation procedure was performed under general anesthesia with endotracheal intubation. Using a cryoballoon ablation catheter, 3-minute occlusive lesion sets were given at each pulmonary vein with additional cryoballoon lesion sets as well as radiofrequency lesion sets given adjacent to the right superior pulmonary vein. Cardiac computed tomography (CT) angiogram was performed instead of endoscopy after negative CT esophagram given the concern for esophageal injury. A 1.4-cm pseudoaneurysm arising from the posterior wall of the left atrium adjacent to the right superior pulmonary vein was seen with multifocal gas within the pericardium, which corresponds to the area where additional lesion sets were given as mentioned above (Figure 1). She was transferred to our tertiary care center and brought to the operating room.

A median sternotomy was performed with institution of CPB with aortic arterial and bicaval venous cannulation. Cardioplegic arrest was performed, and a left atriotomy via the interatrial groove was made. The fistulous connection and associated pseudoaneurysm was visible between the right pulmonary veins. The left atrial tissue was debrided leaving a defect measuring 3.0 x 3.0 cm that was reconstructed with a bovine pericardial patch. The atriotomy was closed, and the patient was weaned from CPB, decannulated, and closed in standard fashion. After repositioning, a right posterolateral thoracotomy was performed, and the sixth intercostal muscle bundle was harvested. The area of the fistula was identified and circumferentially dissected, and the fistula was divided flush to the pericardium with a stapler. This revealed a small esophagus mucosal defect that was closed in 2 layers. The intercostal muscle flap was buttressed in between the esophageal repair and pericardium. The patient’s postoperative course was relatively uncomplicated, and she discharged to acute rehab approximately 2 weeks after her operation and was tolerating intake by mouth 6 weeks after the operation.

### Patient 2

A 78-year-old male patient presented 2 weeks after atrial ablation and left atrial appendage occlusion with dyspnea and abdominal discomfort. His ablation procedure was performed under general anesthesia with endotracheal intubation with lesion sets given at the left atrial anterior free wall and within the left pulmonary vein carina, but full records of his atrial ablation procedure are not available. Imaging was negative for esophageal leak or intracardiac air but demonstrated hydropneumopericardium. A pericardial drain was placed with an initial output of 500 mL of cloudy fluid. He was transferred to our center for management of presumed esophagopericardial fistula.

A repeat CT esophagram confirmed a distal esophageal leak with communication into the pericardial space and a moderate left pleural effusion. A left chest tube was placed and upper endoscopy performed, which demonstrated a 2-cm cratered ulcer 33 cm from the incisions with no apparent full thickness defect. A fully covered esophageal stent was placed over the defect under fluoroscopic guidance and secured with no residual leak on repeat imaging. A CT head was negative for acute intracranial abnormalities. The patient’s diet was advanced, and his drains were removed. The patient was discharged to home on extended antibiotics and antifungals on hospital day 7.

**Figure 2:**
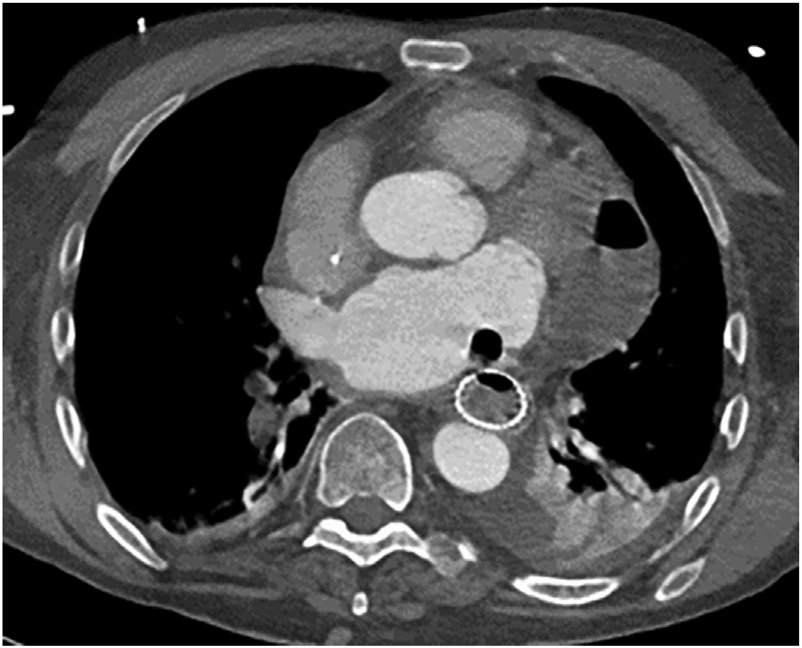
Cardiac computed tomography angiograph demonstrating progression to an atrial esophageal fistula with a complex fluid collection within the pericardium.

The patient re-presented to an outside hospital 2 weeks after discharge in septic shock. After conservative management, he was transferred back to our facility on hospital day 5. CT head and chest demonstrated new multifocal air emboli, small volume subarachnoid hemorrhage, and a persistent esophagopericardial fistula with an increased pericardial gas and fluid collection measuring 7.1 x 4.0 cm but no obvious intracardiac air. A cardiac CT angiogram confirmed intracardiac air indicating progression of the AEF (Figure 2). An interval CT head demonstrated enlargement of his subarachnoid hemorrhage with new midline shift and continued air emboli. After discussion with the family, care was redirected, and the patient expired.

### Patient 3

**Figure 3:**
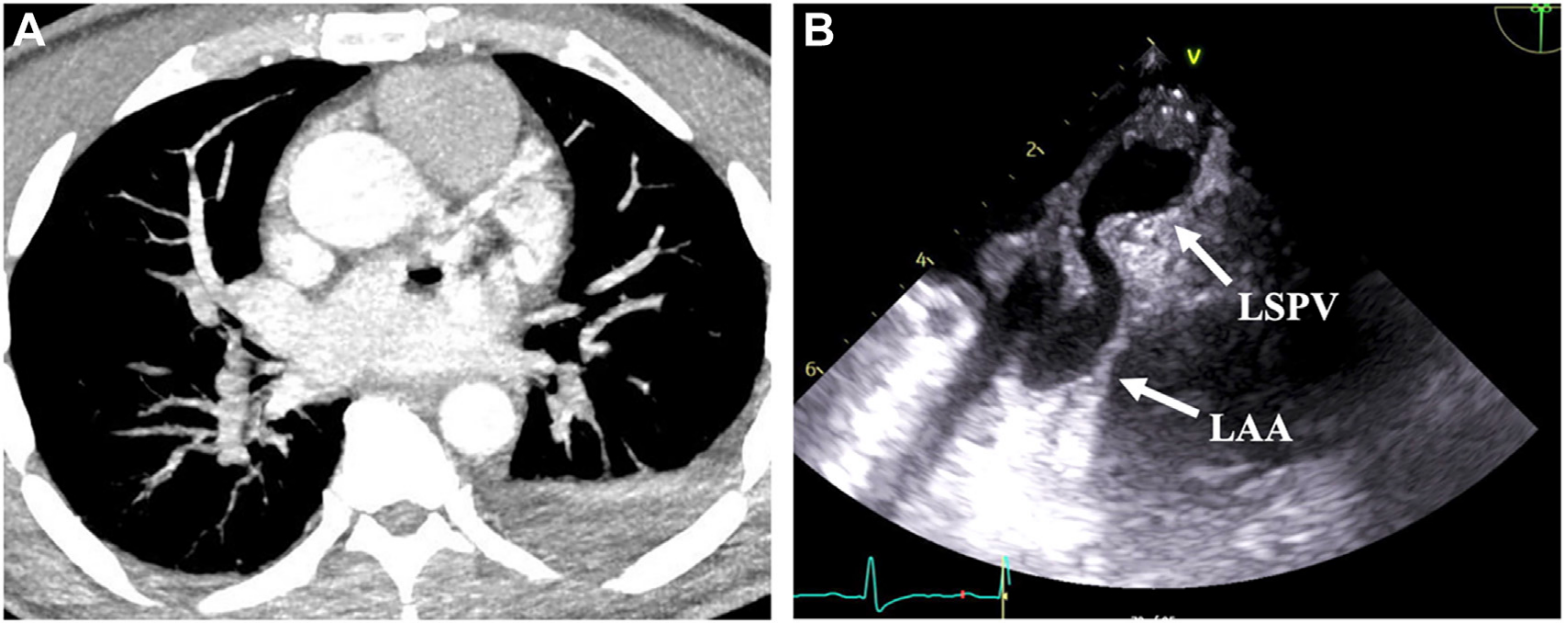
(A) Cardiac computed tomography angiograph demonstrating intracardiac air with complex left atrial mass. (B) Transesophageal echocardiogram demonstrating the atrial mass arising from the posterior atrial wall adjacent to the left superior pulmonary vein (LSPV) extending into the left atrial appendage (LAA).

A 48-year-old male patient with medical history significant for Ehlers-Danlos syndrome presented 6 weeks after an atrial ablation with bacteremia and new-onset neurologic deficits. No records of his atrial ablation procedure are available. He was found to have an acute intraparenchymal hemorrhage and required intubation due to neurologic decline. He was transferred to our facility for further care. The patient developed an intracranial abscess, bilateral cerebral embolic infarcts, and a splenic abscess with no evidence of endocarditis on transesophageal echocardiogram. After undergoing burr hole drainage of his intracranial abscess and percutaneous splenic abscess drainage, the patient had a repeat transesophageal echocardiography for persistent bacteremia, which revealed a mass originating from the left atrial wall near the left superior pulmonary vein. Cardiac CT angiogram demonstrated intracardiac air consistent with an AEF (Figure 3). The patient was taken to the operating room, and, without insufflation, a fully covered esophageal stent was placed under fluoroscopic guidance. Twelve days after stent placement, repeat imaging showed evidence of a persistent AEF, so the patient was taken to the operating room for repair without the use of CPB. The esophageal stent was removed, and a right posterolateral thoracotomy was performed. After identifying the area of concern, the esophagus was encircled with Penrose drain superior and inferior to the fistula, and an endoscopic stapler was fired across the fistula, separating the esophagus from the atrium. The fifth intercostal bundle was harvested as a muscle flap and secured between the atrial and esophageal staple lines, and then the thoracotomy was closed.

The patient experienced a complicated postoperative course including a large volume lower gastrointestinal bleed secondary to an arteriovenous malformation in the distal jejunum. He otherwise required extensive inpatient rehabilitation but was ultimately discharged to an acute rehabilitation center able to tolerate oral intake but with notable cognitive and functional deficiencies.

### Patient 4

**Figure 4:**
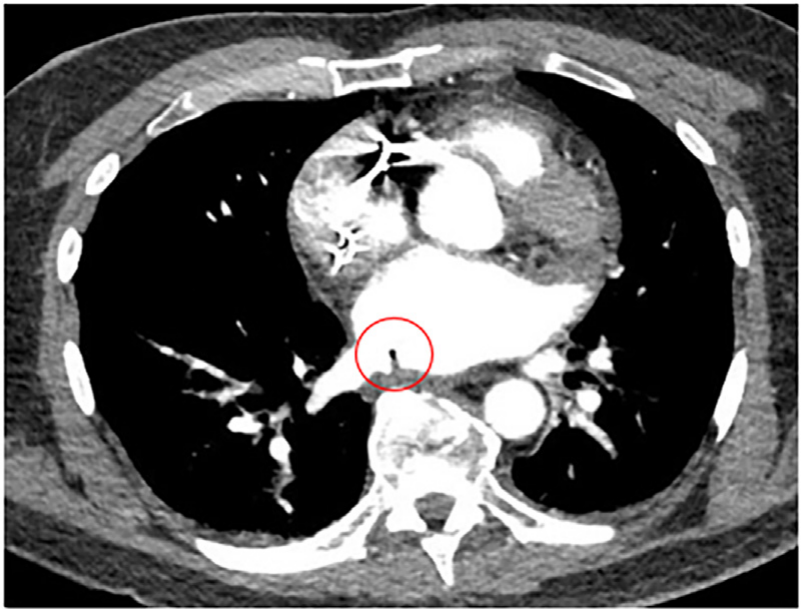
Atrial esophageal fistula (red circle) adjacent to the right inferior pulmonary vein. Reprinted from Felmly LM, Gibney BC. Atrioesophageal fistula from percutaneous ablation for atrial fibrillation. *JTCVS Tech*. 2020;6:167-168. doi:10.1016/j.xjtc.2020.11.020, with permission from The American Association for Thoracic Surgery.

A 59-year-old male patient presented 1 month after an atrial ablation procedure with symptoms of fever, bacteremia, altered mental status, and right-sided hemiparesis. For his ablation procedure, radiofrequency ablation was used, and he was placed under general anesthesia with endotracheal intubation. A temperature probe was utilized, and if esophageal temperature increased 0.5°C, the ablation was stopped until the temperature returned to baseline. The ablation was performed at 40–45 W with reduction in power if in close proximity to the esophagus. Antral pulmonary vein isolation was performed for all 4 pulmonary veins. At the time of admission, a CT head was negative for embolic and hemorrhagic stroke. Further imaging demonstrated intracardiac air and evidence of an AEF at the base of the right inferior pulmonary vein (Figure 4). He was taken emergently for operative repair. A right posterolateral thoracotomy was performed, and the fifth intercostal muscle bundle was harvested for an interposition graft. The fistula was encountered in the suspected location. The patient was placed in the Trendelenburg position, and the fistula was sharply divided. Pledgeted Prolene (Ethicon) sutures were used in a single vertical mattress stitch to close the atrial fistula. The esophagus was debrided down to healthy tissue, and the muscularis opened to reveal the full length of the mucosal injury. The esophageal defect was then repaired in 2 layers. The harvested intercostal muscle was secured between the esophagus and atrium. An anterior and a posterior chest tube were placed, and a flat Jackson Pratt drain was placed adjacent to the esophageal repair. A jejunostomy and gastrostomy tube were then placed via an upper midline laparotomy.

The patient had an overall uncomplicated postoperative course. He had gradual neurologic recovery and discharged to a neurorehabilitation facility with long-tern intravenous antibiotics. His diet was slowly progressed, and at the last surgical clinic visit his gastrostomy and jejunostomy tubes removed.

## Comment

This report represents different strategies used at our institution for the treatment of AEF, including attempted endoscopic management as well as surgical management using 3 distinct operative approaches. The gold standard for treatment is prompt diagnosis and early surgical intervention for repair of the AEF. In a meta-analysis of 85 studies of AEF outcomes, those who underwent surgical repair had a mortality rate of 33%, compared with 65% in the endoscopic treatment group.^5^ In an international registry survey of 118 patients diagnosed with AEF, mortality for surgical repair (51.9%) and endoscopic treatment (56.5%) were much more comparable.^4^ However, it is important to note the ranging risk profile of patients presenting with AEF. Patients undergoing atrial ablation are likely to have preprocedural risk factors, making them high-risk surgical candidates. Data regarding size, location, and extent of AEF are also lacking in these studies. A small focal AEF diagnosed early in a moderate to higher surgical risk patient may be more ideal for an endoscopic and percutaneous therapy compared with open surgery. If the patient is amenable to surgical repair, techniques include thoracotomy and/or sternotomy with or without the use of CPB. The benefit of using a 2-stage approach with the use of CPB is atrial decompression, which allows for a tension-free atrial repair and minimizes risk of air embolism.^3^ However, the patient’s clinical stability may not tolerate such an extensive operation and the hemodynamic consequences of extracorporeal support. In addition, the presence of septic emboli in many patients with AEF put them at high risk for hemorrhagic conversion of ischemic cerebral injuries with high-dose heparin.^6^, ^7^, ^8^

The presence of a left atrial pseudoaneurysm in Patient 1 along with prompt diagnosis in a young otherwise healthy individual led to the decision of using CPB in a 2-stage approach. Left atrial wall pseudoaneurysm is typically a sequela of endocarditis, but the inflammatory state of an AEF and adjacency of the esophagus and left atrial wall likely leads to similar pathophysiology.^9^ To our knowledge, this is the first report of a left atrial pseudoaneurysm resulting from an AEF. For those without a sequela of AEF requiring CPB-assisted repair, an effective strategy could include a right thoracotomy, staple-assisted division of the fistula, 2-layered primary esophageal and atrial repair with tissue flap, wide drainage, and possibly obtaining distal feeding access. Keeping the patient in the Trendelenburg position while dividing the fistula and repairing the atrium under water can help reduce the risk of air entrainment. Consideration should be given to flooding the field with carbon dioxide to further avoid a physiologically serious air embolism.

In conclusion, AEF remains a lethal postprocedure complication that cardiothoracic surgeons will need to address. Based on the outcomes seen in this case series, both a 2-stage operation with use of CPB as well as a one-stage operation through only a right thoracotomy appear to be effective operative approaches for repair. Determination of the optimal approach is beyond the scope of this series, but it is clear that delay in diagnosis can lead to significant morbidity as seen in Patient 3. Prompt diagnosis is a key feature of improving outcomes which will require a joint effort of all stake holders in educating both patients and the greater healthcare community. Patients who have recently undergone atrial ablation should be given strict precautions regarding the new onset of esophageal symptoms, signs of infection, or neurologic changes. If symptoms occur, they are likely to present in the first weeks to months after ablation, making interval follow-up paramount to observe any clinical changes. While specific technical conclusions are outside of the scope of this limited experience, early intervention is crucial, with surgical repair remaining the standard treatment. Endoscopic techniques should be reserved for select patients with prohibitive operative risk.
