# Supplementary material for: Atrial Esophageal Fistulas: A Case Series Demonstrating Three Distinct Operative Approaches with Favorable Outcomes
Source: Ann Thorac Surg Short Rep. 2025 Apr 8;3(3):728–33. doi: 10.1016/j.atssr.2025.03.017 (PMC12559572; doi:10.1016/j.atssr.2025.03.017)
Supplement: Supplementary Table 1 [file mmc1.docx]

**Supplemental Table 1:** Comparison of case presentations, interventions, and post-operative courses

| **Case** | **Case 1** | **Case 2** | **Case 3** | **Case 4** |
| --- | --- | --- | --- | --- |
| **Patient Age** | 47 | 78 | 48 | 59 |
| **Patient Gender** | Female | Male | Male | Male |
| **Risk Factors** | GERD, endotracheal intubation and general anesthesia, additional lesion sets given at the right superior pulmonary vein | Endotracheal intubation and general anesthesia | Ehlers-Danlos Syndrome | Endotracheal intubation and general anesthesia, use of esophageal temperature probe |
| **Time to Presentation** | 3 weeks | 2 weeks | 6 weeks | 4 weeks |
| **Presenting Symptoms** | Fevers, chills | Dyspnea, abdominal pain | Neurological changes | Neurological changes |
| **Type of Ablation** | Cryoballoon and Radiofrequency ablation | Unspecified | Records unavailable | Radiofrequency ablation |
| **Primary Intervention** | Patch repair of atrial defect with CPB via sternotomy; Right thoracotomy with fistula division and primary esophageal repair with ICM buttress | Endoscopic esophageal stent; pericardial drain; tube thoracostomy | Failed esophageal stenting; Right thoracotomy with fistula division and primary esophageal repair with ICM buttress | Right thoracotomy with fistula division and primary esophageal repair with ICM buttress |
| **Time to discharge** | 2 weeks | 1 week | 14 weeks | 4 weeks |
| **Readmission** | No | Yes | No | No |
| **Survival** | Yes | No | Yes | Yes |

GERD: Gastroesophageal Reflux Disease; CPB: Cardiopulmonary Bypass; ICM: Intercostal muscle
